# Resistance of Geosynthetics against the Isolated and Combined Effect of Mechanical Damage under Repeated Loading and Abrasion

**DOI:** 10.3390/ma12213558

**Published:** 2019-10-30

**Authors:** Filipe Almeida, David Miranda Carlos, José Ricardo Carneiro, Maria de Lurdes Lopes

**Affiliations:** Construct-Geo, Faculty of Engineering, University of Porto, Rua Dr. Roberto Frias, 4200-465 Porto, Portugal; davidmc@fe.up.pt (D.M.C.); rcarneir@fe.up.pt (J.R.C.); lcosta@fe.up.pt (M.d.L.L.)

**Keywords:** geosynthetics, geotextiles, geogrids, degradation, mechanical damage under repeated loading, abrasion, reduction factors, synergisms

## Abstract

The behaviour of materials used for developing engineering structures should be properly foreseen during the design phase. Regarding geosynthetics, which are construction materials used in a wide range of engineering structures, the installation on site and the action of many degradation agents during service life may promote changes in their properties, endangering the structures in which they are applied. The evaluation of the damage suffered by geosynthetics, like installation damage or abrasion, is often carried out through laboratory tests. This work studied the behaviour of five geosynthetics (three geotextiles and two geogrids) after being individually and successively exposed to two degradation tests: mechanical damage under repeated loading and abrasion. The short-term mechanical and hydraulic behaviours of the geosynthetics were analysed by performing tensile tests and water permeability normal to the plane tests. Reduction factors were determined based on the changes occurred in the tensile strength of the geosynthetics. Findings showed that mechanical damage under repeated loading and abrasion tended to affect the mechanical and hydraulic properties of the geosynthetics and that the reduction factors calculated according to the traditional method may not be able to represent accurately the damage suffered by the materials when exposed successively to the degradation mechanisms.

## 1. Introduction

The design of an engineering structure includes the use of safety factors to properly safeguard the occurrence of permanent and variable actions. For instance, safety factors should be applied to the properties of a construction material to prevent the possible occurrence of adverse deviations of those properties over time.

The use of geosynthetics in engineering structures has become a common practice in the last decades. Their high versatility and efficiency, associated to the ease of installation and relatively low cost, make them suitable construction materials to perform many different functions, for example, protection, reinforcement, separation, filtration, drainage and fluid barrier [1,2]. Geosynthetics can be used in a wide range of applications such as embankments, roads and railways infrastructures, retaining walls, erosion control or coastal protection, in which they may be exposed to the action of many degradation agents. Creep, abrasion, high temperatures, oxygen, atmospheric agents (e.g., solar radiation) and chemical substances (e.g., acids or alkalis) are examples of agents that may affect the durability of geosynthetics [1,3]. An extended exposure to these agents can promote relevant changes in the properties of geosynthetics, compromising their performance and reducing their lifetime [1,3].

The installation procedures may also affect the properties of geosynthetics and, consequently, have a negative impact on their performance. The handling of geosynthetics and the placement and compaction of filling materials over them may induce damage (predominantly mechanical) on their structure [1,3]. Indeed, in a wide range of applications, it is during the installation process that geosynthetics are submitted to the highest mechanical stresses [3,4]. The installation procedures may induce cuts in components, tears, holes and, consequently, a reduction in mechanical resistance of geosynthetics [5,6]. The survivability of these materials during installation is highly dependent on different factors, such as the physical properties of geosynthetics, the characteristics of the soils, the compaction energy and the use, or not, of adequate installation procedures [4,6,7,8]. In addition to the types of damage mentioned before, the installation procedures may also provoke abrasion on geosynthetics due to the mobilization of frictional forces in the interface between these materials and the contacting aggregates. For example, abrasion is prone to occur during the placement and compaction of filling materials in road construction or during the sand filling of geosystems (e.g., geocontainers, geobags or geotubes) for coastal protection. In some applications (such as roads or railways infrastructures), the occurrence of abrasion is not restricted to the installation phase. Indeed, due to the occurrence of cyclic loads over time (e.g., resulting from vehicle traffic), geosynthetics may also experience abrasion. The effect of the installation process on the properties of geosynthetics must be known and accounted for at the design phase. Depending on the application, the effect of abrasion (if considered a relevant degradation mechanism) has also to be taken into account.

The level of damage imposed by the installation procedures to geosynthetics can be simulated through field tests (which require the use of heavy equipment and significant human and financial resources) or by conducting laboratory tests (which try to simulate the in situ damaging actions). The standard EN ISO 10722 [9] describes a laboratory method to induce mechanical damage under repeated loading on geosynthetics. Many authors have used this method to estimate the damage suffered by geosynthetics during installation [8,10,11], while others tried to establish correlations between this method and the damage occurred under real installation conditions [6,12]. Pinho-Lopes and Lopes [6] concluded that the laboratory damage (induced by the method presented in ENV ISO 10722-1 [13], which was superseded by EN ISO 10722 [9]) can be more severe to geosynthetics in comparison with field installation damage. The effect of abrasion on geosynthetics has not been as studied as installation damage. However, there is also a method (described in EN ISO 13427 [14]) for evaluating the resistance of geosynthetics against this degradation mechanism. This method can induce relevant damage on those materials, such as cuts in components, splitting or disintegration, significantly affecting their properties [15,16].

The evaluation of the damage suffered by geosynthetics during the degradation tests is often accomplished by analysing the changes occurred in their mechanical properties [4,5,6,8,15,16]. In addition, the changes occurred in their hydraulic properties have also been assessed in some works [8,16,17]. Mechanical characterisation (after the degradation tests) often includes the determination of the tensile [4,5,6,8,15,16] (especially relevant for geogrids, which are used for soil reinforcement) and puncture [8,15] behaviours of the geosynthetics. Hydraulic characterisation (for example, by water permeability tests) is important when considering the use of geosynthetics for performing filtration or drainage functions.

The design values of geosynthetics must take into account the level of degradation that those construction materials are going to experience over time. For that purpose, partial reduction factors are often used, each representing the effect (known or estimated) of one, or more, degradation agents [18,19,20]. For example, for reinforcement applications, ISO/TR 20432 [18] considers the use of four partial reduction factors for affecting the tensile strength of geosynthetics, accounting for the effects of installation damage, creep, weathering, and chemical and biological agents. The partial reduction factors are usually obtained in isolation, discarding the possible interactions that may occur between the degradation agents [21]. The global reduction factor (used in the design) is traditionally obtained by multiplying the partial reduction factors. However, the multiplication of two, or more, partial reduction factors obtained in isolation does not always represent accurately the combined effect of the degradation agents [15,21]. Literature review has revealed the existence of interactions between the different degradation agents. When analysing the action of installation damage and creep, Allen and Bathurst [22], Greenwood [23] and Cho et al. [24] concluded that the traditional method to obtain the reduction factors for the combined action of those degradation agents might be conservative. By contrast, Carneiro et al. [21] and Carneiro et al. [25] showed that the existence of interactions between chemical degradation agents can result in inaccurate reduction factors (by underestimation) if the traditional method is followed to obtain the reduction factors for the combined action of those agents. Interactions have also been found between mechanical damage under repeated loading and abrasion, which also led to inaccurate reduction factors when using the traditional method [15,16].

This work focused on the evaluation of the effect of mechanical damage under repeated loading and abrasion on geosynthetics, contributing to improve the knowledge about these two degradation mechanisms. Five geosynthetics (a woven and two nonwoven geotextiles, and two geogrids) were submitted individually and successively to mechanical damage under repeated loading and abrasion tests. The damage induced by these tests on geosynthetics was assessed by performing tensile tests and water permeability normal to the plane tests (the latter, only for the geotextiles). Based on the changes occurred in the tensile strength of the geosynthetics, reduction factors were determined. The reduction factors determined by the traditional method (multiplication of reduction factors obtained in isolation for each degradation mechanism) were compared with those obtained in the successive exposures to mechanical damage under repeated loading and abrasion. The results showed that the action of the degradation mechanisms tended to affect the mechanical and hydraulic behaviours of the geosynthetics and that the traditional method (for the determination of reduction factors) was not able to represent accurately the combined effect of mechanical damage under repeated loading and abrasion. Indeed, the predicted reduction factors tended to be lower in comparison with those found in the successive exposures to both degradation mechanisms.

## 2. Materials and Methods

### 2.1. Geosynthetics

Five geosynthetics were used in the experimental work: a woven geotextile made from high-density polyethylene filaments, two nonwoven geotextiles manufactured with polypropylene fibres, and two woven geogrids, one made from polypropylene fibres and the other from polyester fibres. Table 1 provides information about their mass per unit area and thickness, which were determined according, respectively, to standards EN ISO 9864 [26] and EN ISO 9863-1 [27], and also displays the tensile strength of each geosynthetic, obtained in accordance with EN ISO 10319 [28]. The designations ascribed to the geotextiles give information about their structure (“W” and ”NW” for, respectively, woven and nonwoven) and nominal mass per unit area (values defined by the manufacturers). Regarding geogrids, the numbers following the abbreviation “GG” are a reference to their nominal tensile strengths. The sampling and preparation of specimens was made in accordance with the instructions displayed in standard EN ISO 9862 [29].

### 2.2. Degradation Tests

The geosynthetics were submitted to two types of degradation tests: mechanical damage under repeated loading and abrasion. The materials were, in a first stage, individually exposed to each degradation test (single exposures to mechanical damage under repeated loading and abrasion). Then, the geosynthetics were successively submitted to mechanical damage under repeated loading and abrasion (multiple exposure). For each case were tested five specimens of each geosynthetic.

**Figure 1:**
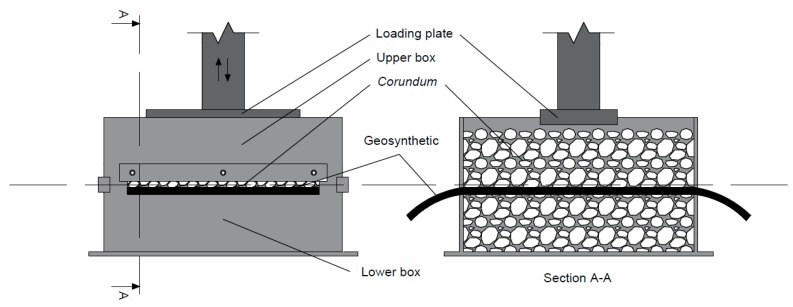
Schematic representation of the equipment for mechanical damage under repeated loading tests.

The mechanical damage under repeated loading tests (hereinafter MD tests) were carried out according to EN ISO 10722 [9]. These tests, which were conducted on a laboratory prototype (Figure 1) developed at the Faculty of Engineering of the University of Porto (a full description of the equipment is available in [30]), comprised the placement of a sample of geosynthetic between two layers of *corundum* (a synthetic aggregate from aluminium oxide) of the same height (75 mm) that were installed in the lower and upper boxes of equal dimensions (length and width of 300 mm, and height of 87.5 mm), followed by the application of a cyclic loading between (5.0 ± 0.5) kPa and (500 ± 10) kPa at a frequency of 1 Hz for 200 cycles, by means of a loading plate (length and width of, respectively, 200 mm and 100 mm). It is worthy to mention that the layer of *corundum* added to the lower box was placed in two sublayers of the same height (37.5 mm each), both submitted to a compaction process, for 60 s, at a pressure of (200 ± 2) kPa throughout the entire box area. The layer of *corundum* (height of 75 mm) placed in the upper box was not submitted to a compaction process.

**Figure 2:**
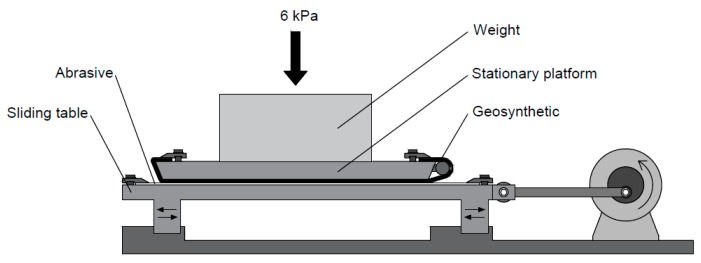
Schematic representation of the equipment for abrasion tests.

The abrasion tests were carried out on a prototype (Figure 2) developed at the aforementioned institution in accordance with EN ISO 13427 [14]. These tests consisted of attaching a sample of geosynthetic to a stationary platform placed above a sliding table, in which was settled a sheet of a P100 abrasive. After assembling all components, the sliding table was moved along a horizontal axis (cyclic uniaxial movement), fostering the rubbing of the samples by the abrasive, under a controlled pressure of 6 kPa for 750 cycles. In each cycle, the abrasive passed twice through the geosynthetic (a back-and-forth linear motion).

### 2.3. Damage Evaluation of The Geosynthetics

#### 2.3.1. Visual Inspection

A qualitative evaluation, by visual inspection, of the damage induced by the degradation tests on the geosynthetics was carried out prior to tensile and water permeability normal to the plane tests. The visual inspection contributed to understand the type and level of deterioration suffered by the geosynthetics. This assessment provides an idea about potential undesirable changes that may occur in materials properties, which would be (or not) corroborated with the tensile and water permeability normal to the plane tests.

#### 2.3.2. Tensile Tests

A Lloyd Instruments testing machine (model LR10K Plus) fitted with a load cell of 10 kN was used to conduct the tensile tests on the geosynthetics (specimens in the machine direction of production). The tensile tests were carried out in accordance with EN ISO 10319 [28] at a displacement rate of 20 mm·min^−1^, and the data resulting from those tests allowed the determination of the tensile strength (T, in kN·m^−1^) and the elongation at maximum load (E_ML_, in %) of the geosynthetics. The values displayed in this work correspond to the arithmetic mean of five tested specimens and are presented with 95% confidence intervals determined according to Montgomery & Runger [31]. The retained tensile strengths (in %) of the materials, which resulted from the quotient between the tensile strengths of the damaged and undamaged samples, were determined and used as a tool to monitor the variations of the tensile strengths.

#### 2.3.3. Water Permeability Normal to the Plane Tests

The water permeability of the geotextiles (with a water flow normal to the plane) was evaluated according to the constant head method displayed in EN ISO 11058 [32]. The method consisted of submitting a geotextile specimen with a diameter of 125.0 mm (the useful diameter of each specimen was 83.5 mm) to a unidirectional flow of water normal to the plane under different constant head losses: 70, 56, 42, 28 and 14 mm. Five specimens for undamaged samples and the same number for each of the single and multiple degradation tests mentioned in Section 2.2 were tested.

Targeting the desired outcome of these tests, which was the velocity index for a head loss of 50 mm (*V_H50_*, in mm·s^−1^), the determination of the flow velocity (*v*_20_, in mm·s^−1^) was carried out for each of the five head losses, which was given by:

(1) $$v_{20}=(V\;R_{T})/\left(A\;t\right),$$

where *V* and *t* are, respectively, the water volume measured (in mm^3^) and the time to achieve this volume (in seconds), *R_T_* is the correction factor to the water temperature (which was determined according to Annex A of EN ISO 11058 [32]) and *A* is the exposed specimen area (5476 mm^2^). After plotting the five head losses against the respective *v*_20_ of each specimen, a quadratic regression curve passing through the origin was adjusted, and the *V_H50_* was calculated by interpolation. The values of *V_H50_* resulting from these tests are also presented with 95% confidence intervals determined according to Montgomery & Runger [31].

### 2.4. Reduction Factors

Based on the changes occurred in the tensile strength of the geosynthetics after the degradation tests, reduction factors (RFs) were calculated according to the following equation:

(2) $$\mathrm{RF}=T_{\mathrm{Undamaged}}/T_{\mathrm{Damaged}},$$

where T_Undamaged_ and T_Damaged_ are, respectively, the tensile strength of the geosynthetics before and after the degradation tests.

According to the traditional method, the RF used to account for the combined effect of two, or more, degradation agents is obtained by multiplying the RFs resulting from the single exposures to each degradation agent. Therefore, for the combined effect of mechanical damage under repeated loading and abrasion, the traditional RF (RF_MD+ABR TRAD_) is given by:

(3) $${\mathrm{RF}}_{\mathrm{MD}+\mathrm{ABR}\;\mathrm{TRAD}}={\mathrm{RF}}_{\mathrm{MD}}\times {\mathrm{RF}}_{\mathrm{ABR}},$$

where RF_MD_ and RF_ABR_ are, respectively, the RFs resulting from the isolated action of mechanical damage under repeated loading and abrasion.

It should be emphasized that the RFs obtained in this experimental campaign are an outcome of standard tests that may not represent the conditions that the materials are going to experience during their installation on site and during service life. Moreover, the correspondence between the degradation conditions resulting from the standard tests and those observed in field, which differ from project to project, was not carried out since it was not a goal of this work. Indeed, the laboratory standard tests impose, in many cases, more severe scenarios than in situ conditions. Therefore, the RFs presented in this work are not suitable for design purposes. Their determination was intended to compare the methods used for obtaining the RFs for the combined action of mechanical damage under repeated loading and abrasion: traditional method vs. successive exposure to both degradation mechanisms.

## 3. Results and Discussion

### 3.1. Visual Inspection

**Figure 3:**
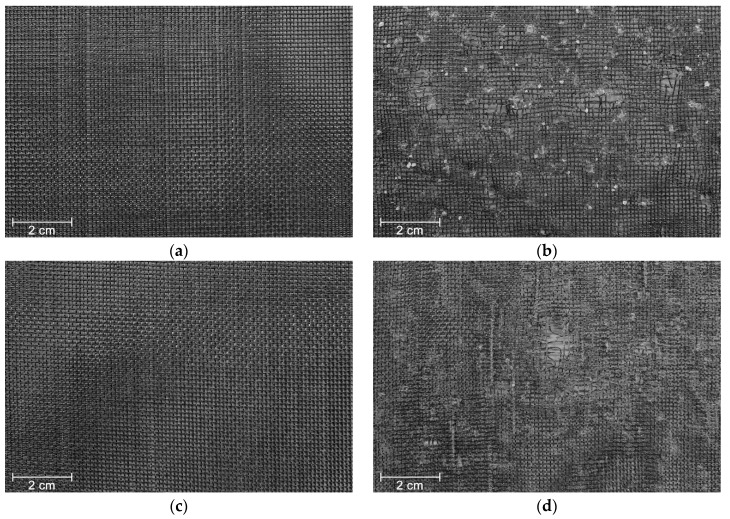
Visual inspection of geotextile W180: (**a**) undamaged sample; (**b**) after mechanical damage under repeated loading; (**c**) after abrasion; (**d**) after successive exposure to mechanical damage under repeated loading and abrasion.

The visual inspection performed on geotextile W180 contributed to understand the level of deterioration induced by the degradation tests. The MD tests caused filaments cutting (Figure 3b), which readily indicated potential losses in tensile strength. In addition, the filaments were scratched, and it was observed the presence of very small particles of *corundum* trapped in the woven structure (particles resulting from the splintering of *corundum* during the MD tests). By contrast, the abrasion tests did not provoke substantial damage on geotextile W180, since only scratched filaments were observed (the scratches cannot be observed at the magnification of Figure 3c). After the successive exposure to both degradation tests, the damage found on geotextile W180 was similar to the damage observed after the single exposure to the MD tests (Figure 3d).

**Figure 4:**
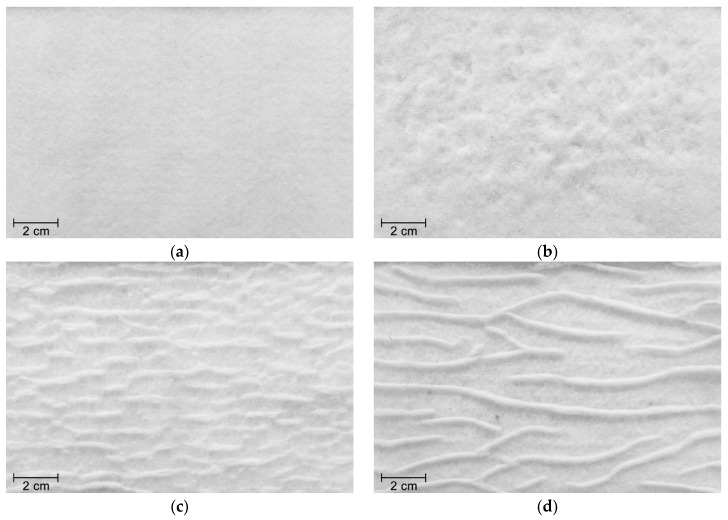
Visual inspection of geotextile NW300: (**a**) undamaged sample; (**b**) after mechanical damage under repeated loading; (**c**) after abrasion; (**d**) after successive exposure to mechanical damage under repeated loading and abrasion.

The degradation tests led to different types of damage on the nonwoven structure of geotextile NW300. The MD tests induced punctures and fibres cutting (Figure 4b). As observed for geotextile W180, geotextile NW300 had also some *corundum* particles imprisoned on its structure. Regarding abrasion, besides the fibres cutting, clusters of fibres were formed on the surface layer of the geotextile, perpendicularly to the movement of the sliding table (Figure 4c). The exposure of geotextile NW300 to both degradation tests also resulted in the formation of clusters of fibres (in this case, less abundant but larger). In addition, some holes with small diameter were observed after the abrasion tests, probably due to the action of *corundum* particles trapped in the nonwoven structure (Figure 4d).

The outcomes of exposing geotextile NW500 to the different degradation agents were relatively similar to those found in geotextile NW300. Nevertheless, the punctures and fibres cutting caused by the MD tests on geotextile NW500 were less severe in comparison with geotextile NW300. In addition, no holes were observed after the successive exposure to mechanical damage under repeated loading and abrasion (regarding the single exposure to abrasion, no relevant differences were found on the degradation suffered by the geotextiles).

**Figure 5:**
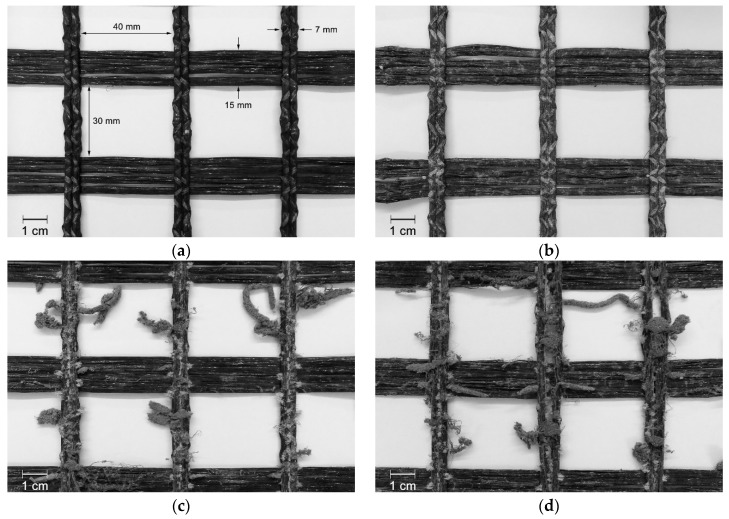
Visual inspection of geogrid GG40I: (**a**) undamaged sample; (**b**) after mechanical damage under repeated loading; (**c**) after abrasion; (**d**) after successive exposure to mechanical damage under repeated loading and abrasion.

The geogrid GG40I apparently did not suffer significant damage during the MD tests (Figure 5b). Its crossbars (which had a low, or inexistent, impact on tensile strength, since the geogrids were tested in the machine direction of production) were scratched and had attached some particles arising from the splintering of *corundum*. By contrast, the longitudinal bars (resistant elements of the geogrids in the machine direction of production) have emerged practically unscathed from the MD tests. The abrasion tests were more aggressive to geogrid GG40I compared with the MD tests: its longitudinal bars were highly affected (many fibres were cut and detached from the bars, which resulted in a reduction of their thickness) (Figure 5c). The damage detected on the geogrid’s crossbars was virtually none. The longitudinal bars, located at a distinct plane, were directly exposed to the action of the abrasive, this way protecting the crossbars. The successive exposure to both degradation mechanisms resulted in a deterioration of the longitudinal bars relatively similar to the single exposure to abrasion (Figure 5d). The crossbars had some scratches, but, apparently, their fibres were not cut.

**Figure 6:**
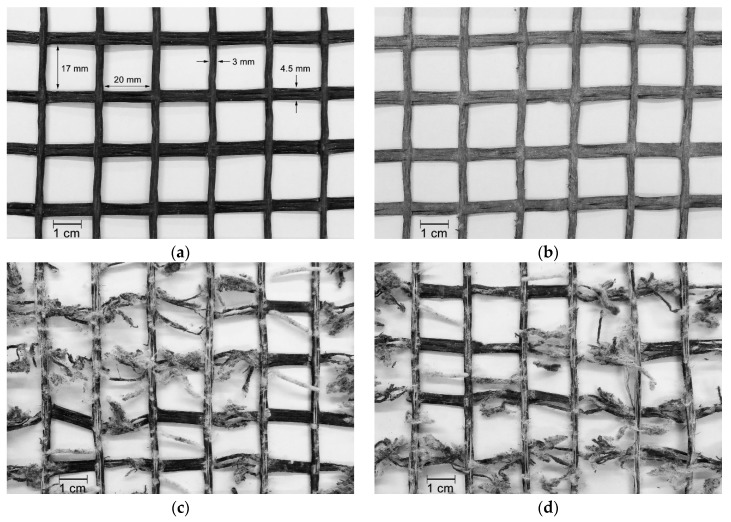
Visual inspection of geogrid GG40II: (**a**) undamaged sample; (**b**) after mechanical damage under repeated loading; (**c**) after abrasion; (**d**) after successive exposure to mechanical damage under repeated loading and abrasion.

The MD tests did not appear to induce relevant damage on geogrid GG40II, as showed in Figure 6b. By contrast, the abrasion tests promoted the destruction of a relevant number of both longitudinal bars and crossbars, indicating the existence of relevant changes in the tensile behaviour of geogrid GG40II (Figure 6c). With regard to the successive exposure to both degradation mechanisms, the level of visible damage of geogrid GG40II was similar to the single exposure to abrasion (Figure 6d).

### 3.2. Tensile Properties

#### 3.2.1. Woven Geotextile

The tensile properties of geotextile W180, before and after being submitted to the degradation tests, are exhibited in Table 2. As expected, considering the visual inspection, the single exposure to mechanical damage under repeated loading caused a higher loss in tensile strength, compared with abrasion. The differences are appreciable, being the retained tensile strengths after the single exposures to mechanical damage under repeated loading and abrasion of, respectively, 35.6% and 86.1%. The action of *corundum* particles, which had a rough texture and an angular shape, was too aggressive to the geotextile, causing filaments cutting, which led to the deterioration of the tensile behaviour. Regarding the abrasion tests, although no significant damage was detected besides scratched filaments, a slight decrease occurred in the tensile strength of geotextile W180 (loss of 13.9%). Contrary to what happened during the MD tests, the rigid structure of the geotextile had a relatively good resistance against the action of the abrasive, avoiding filaments cutting, hence resulting in lower changes in tensile strength. Similarly to tensile strength, elongation at maximum load also suffered relevant decreases after the single exposures to mechanical damage under repeated loading and abrasion (decrease significantly more pronounced after the MD tests).

The successive exposure to both degradation mechanisms led to a decrease in tensile strength higher than that occurred after the single exposure to the MD tests (retained tensile strengths of 27.8% and 35.6%, respectively). In addition to the already highly damaged structure, the presence of fine particles of *corundum* may have contributed to induce additional damage on geotextile W180 during the abrasion tests. The elongation at maximum load has also decreased in comparison with the single exposures to the degradation tests.

#### 3.2.2. Nonwoven Geotextiles

The nonwoven geotextiles NW300 and NW500, whose tensile properties are displayed in Table 3, were distinctively affected by the degradation mechanisms in terms of induced level of damage. Geotextile NW500 showed to be more resistant against mechanical damage under repeated loading than geotextile NW300, since its retained tensile strength was appreciably higher (86.0% and 69.8%, respectively). These were expected results considering the damage detected by visual inspection (the punctures and fibres cutting were more pronounced in geotextile NW300 than in geotextile NW500). Both geotextiles had a higher resistance against abrasion, in comparison with mechanical damage under repeated loading. Indeed, the retained tensile strengths of geotextiles NW300 and NW500 after abrasion were, respectively, 86.2% and 96.0%. Contrary to what might be expected, the clusters of fibres formed on the surface layer of the geotextiles did not lead to considerable losses in tensile strength.

Regarding the successive exposure to both degradation mechanisms, the results showed that this scenario led to the highest decrease in the tensile strength of geotextile NW300 (retained tensile strength of 50.6%). By contrast, the decrease in tensile strength observed for geotextile NW500 was quite similar to that observed after the single exposure to mechanical damage under repeated loading (retained tensile strengths of, respectively, 86.0% and 84.5%). Like tensile strength, the elongation at maximum load of both geotextiles also decreased after the multiple exposures, being the differences more pronounced for geotextile NW300.

In general, geotextile NW500 had a higher resistance against degradation (lower deterioration of tensile behaviour) than geotextile NW300. This shows the existence of an effect of mass per unit area on the survivability of the geotextiles during the degradation tests. Indeed, the increase of mass per unit area resulted in a better resistance against the damaging actions.

#### 3.2.3. Geogrids

As noticed for the geotextiles, relevant changes were also observed in the tensile behaviour of the geogrids after the single- and multiple-exposure degradation tests (Table 4). The MD tests did not provoke relevant changes in the tensile strength of geogrid GG40I (retained tensile strength close to 100%). The low deterioration of geogrid GG40I can be explained by the robustness of its longitudinal bars (resistant elements), which had a good resistance against mechanical damage under repeated loading (as mentioned in Section 3.1, no damage was observed in geogrid GG40I after the MD tests). By contrast, and as expected, the abrasion tests led to a relevant reduction in tensile strength (loss of 63.1%). This decrease of the tensile strength of geogrid GG40I can be explained by the deterioration occurred in its longitudinal bars (as illustrated in Figure 5c).

The geogrids GG40I and GG40II suffered similar reductions in tensile strength (63.1% and 63.6%, respectively) after the abrasion tests, but had very distinct behaviours after the MD tests. With respect to the latter, geogrid GG40II was significantly more affected than geogrid GG40I (retained tensile strengths of, respectively, 59.7% and 98.4%). Despite the absence of visible defects (Figure 6b), the damaging actions imposed during the MD tests might have provoked relevant deterioration on the woven structure of geogrid GG40II. It is important to mention that the longitudinal bars of geogrid GG40II were not as thicker as those from geogrid GG40I, making them less resistant against the cyclic loads induced during the MD tests. Regarding abrasion, the reduction found in the tensile strength of geogrid GG40II can be ascribed to the extensive degradation occurred in its longitudinal bars (as illustrated in Figure 6c). The single exposure degradation tests also led to changes in the elongation at maximum load of geogrids GG40I and GG40II (more pronounced changes after abrasion).

The multiple exposure to both degradation mechanisms led to quite similar losses in the tensile strength of geogrid GG40I compared with the single exposure to abrasion (retained tensile strengths of 33.3% and 36.9%, respectively). It is worthy to remember that the tensile strength of geogrid GG40I was practically unaffected by the MD tests, which explains the proximity (in terms of losses of tensile strength) between the single exposure to abrasion and the multiple exposure to mechanical damage under repeated loading and abrasion. Regarding geogrid GG40II, the combination of the degradation mechanisms provoked the highest reduction in its tensile strength (loss of 81.1%). In this case, and contrary to what happened for geogrid GG40I, the samples exposed to abrasion had already suffered some degradation during the MD tests (indicated by the loss of 40.3% in tensile strength).

The results showed that, although having similar nominal tensile strengths, geogrid GG40II was less resistant to the degradation mechanisms than geogrid GG40I. The geogrid GG40I had a more robust structure (with more resistant longitudinal bars), which resulted in a higher resistance against degradation. Indeed, the larger and thicker bars of geogrid GG40I had a higher survivability against the damaging actions. Both geogrids are used for reinforcement, but the higher resistance against degradation of geogrid GG40I makes it more suitable than geogrid GG40II to effectively perform that function (considering only the actions of the degradation mechanisms under study). Nevertheless, previous research indicated that extruded geogrids can be more resistant against mechanical damage under repeated loading and abrasion, in comparison with woven geogrids [16].

### 3.3. Hydraulic Properties

Considering the values of the *V_H50_* (Table 5), the water permeability of the undamaged samples of geotextile NW300 was higher compared with geotextile NW500. This can be explained by the lower mass per unit area and thickness of geotextile NW300, which facilitated the water flow through its nonwoven structure. The water permeability of the geotextiles suffered some relevant changes after the single- and multiple-exposure degradation tests (these changes were not the same, either for both geotextiles, or for the different degradation agents) (Table 5).

The MD tests did not induce significant changes in the water permeability of geotextile NW300, only a slight increase in *V_H50_* (the punctures and fibres cutting found in the nonwoven structure were not enough to modify the hydraulic behaviour). By contrast, the abrasion tests (single exposure) led to an increase of 26.8% in *V_H50_*. The detachment of the surface layer of geotextile NW300 (due to the cut of fibres and formation of clusters of fibres—Figure 4c) resulted in a higher water flow through the nonwoven structure. Regarding the successive exposure to mechanical damage under repeated loading and abrasion, it was noticed an increase of 47.1% in *V_H50_*. In this case, the surface layer of the geotextile was significantly more affected compared with the single exposure to abrasion (Figure 4d). Indeed, the detachment of fibres was more pronounced (discarding the clusters of fibres, a reduction was found in the thickness of the nonwoven structure), which facilitated the water flow. The small holes detected in geotextile NW300 may also have contributed to the increase observed in the *V_H50_*.

Contrary to what happened for geotextile NW300, the *V_H50_* of geotextile NW500 had no relevant changes after the single- and multiple-exposure degradation tests. This is consistent with the higher survivability of geotextile NW500 (already referred in Section 3.2.2), indicating once again that the increase of mass per unit area resulted in a higher resistance against degradation. This way, geotextile NW500 had a higher capability to maintain its hydraulic behaviour compared with geotextile NW300, seeming to indicate that its filtration abilities were not significantly affected when exposed to the damaging actions.

### 3.4. Reduction Factors

**Figure 7:**
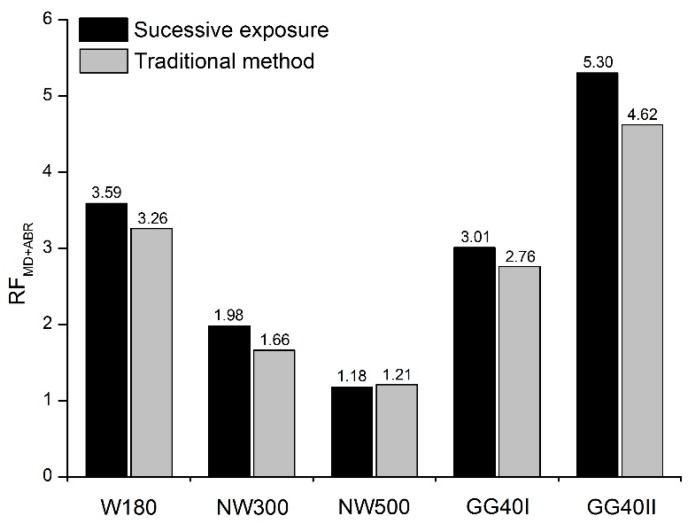
Comparison between the RF_MD+ABR_ found in the successive exposure to mechanical damage under repeated loading and abrasion with those obtained by the traditional method for the combined effect of both degradation mechanisms.

The comparison between the RFs calculated by the traditional method (i.e., by multiplying the RFs resulting from the single exposures to each degradation mechanism (Table 6)) and those obtained through the successive exposure to both degradation mechanisms can be found in Figure 7.

With exception for geotextile NW500, the RFs obtained in the successive exposure to mechanical damage under repeated loading and abrasion tended to be slightly higher than those determined by the traditional method for the combined effect of both degradation mechanisms. For geotextiles W180 and NW300, the traditional method led, respectively, to RFs 9.2% and 16.2% lower than those found in the successive exposure to the degradation mechanisms. Regarding geogrids GG40I and GG40II, the RFs determined by the traditional method were, respectively, 8.3% and 12.8% lower compared with their counterparts resulting from the multiple exposures. Contrary to the previous geosynthetics, the difference between the RFs found for geotextile NW500 was almost negligible (difference of 2.5%).

The higher RFs found in the multiple exposures to mechanical damage under repeated loading and abrasion indicated that the traditional method may not be able to represent accurately (by underestimating) the combined effect of both degradation mechanisms. Dias et al. [15] and Rosete et al. [16] attained similar conclusions for other geosynthetics. An inaccurate determination of RFs may lead to a premature failure of geosynthetics during their service life and, consequently, affect the durability of the structures in which they are installed. The lower and inaccurate RFs obtained by the traditional method can be ascribed to the existence of interactions between the degradation agents, which that method may not able to account for. Therefore, when designing with geosynthetics, it is important to take into consideration the interactions (synergisms) that may occur between their degradation agents. This must be performed case by case, considering the particular conditions and requirements of each engineering project.

## 4. Conclusions

This work evaluated the damage suffered by five geosynthetics after being individually and successively exposed to two degradation mechanisms: mechanical damage under repeated loading and abrasion. The results showed that the single exposures of the materials to mechanical damage under repeated loading and abrasion led, in general, to losses in tensile strength. However, the woven and nonwoven geotextiles were more affected by the mechanical damage under repeated loading tests, whereas the woven geogrids experienced higher tensile strength losses after being exposed to abrasion. The most adverse scenario to all geosynthetics (i.e., where higher tensile strength losses occurred) was the successive exposure to both degradation mechanisms.

The isolated and combined actions of mechanical damage under repeated loading and abrasion led to changes in the water permeability behaviour normal to the plane of the nonwoven geotextile with the lowest mass per unit area (325 g·m^−2^). By contrast, the geotextile with higher mass per unit area (476 g·m^−2^) had no relevant changes in its hydraulic behaviour. Besides the previous effect, mass per unit area also had a key influence in the tensile behaviour of the nonwoven geotextiles. Indeed, the geotextile with higher mass per unit area was significantly less affected by the degradation tests (higher survivability).

Finally, the reduction factors determined by the traditional method for the combined effect of mechanical damage under repeated loading and abrasion tended to be lower than those found in the successive exposure to both degradation mechanisms. This shows that the traditional method may not be representing correctly the combined effect of the degradation mechanisms, not being able to account for the interactions occurred between them. It is important to refer that the reduction factors obtained in this work resulted from particular degradation conditions (which may not correspond to field conditions) and, therefore, it is not reasonable nor advisable the use of these reduction factors for design purposes.

## Figures and Tables

**Table 1:** Main properties of the geosynthetics.

Geosynthetic	Type	µ_A_ (g·m^−2^) ^1^	*t* (mm) ^2^	T (kN.m^−1^) ^3^
W180	Woven geotextile	193 (± 3)	0.84 (± 0.01)	24.80 (± 0.62)
NW300	Nonwoven geotextile	325 (± 11)	3.83 (± 0.09)	23.44 (± 1.33)
NW500	Nonwoven geotextile	476 (± 12)	4.68 (± 0.11)	33.61 (± 2.33)
GG40I	Woven geogrid	-	-	44.36 (± 1.02)
GG40II	Woven geogrid	-	-	39.83 (± 1.74)

^1^ Mass per unit area; ^2^ Thickness; ^3^ Tensile strength. (95% confidence intervals in brackets.)

**Table 2:** Tensile properties of the woven geotextile W180.

Degradation Test	T (kN·m^−1^)	E_ML_ (%)
Undamaged	24.80 (± 0.62)	52.7 (± 4.5)
MD	8.84 (± 0.70)	17.2 (± 1.6)
Abrasion	21.36 (± 0.56)	34.2 (± 1.6)
MD + Abrasion	6.90 (± 0.61)	13.2 (± 1.1)

MD: mechanical damage under repeated loading. (95% confidence intervals in brackets.).

**Table 3:** Tensile properties of the nonwoven geotextiles NW300 and NW500.

Degradation Test	NW300		NW500	
	T (kN·m^−1^)	E_ML_ (%)	T (kN·m^−1^)	E_ML_ (%)
Undamaged	23.44 (± 1.33)	138.4 (± 13.8)	33.61 (± 2.33)	135.9 (± 17.6)
MD	16.36 (± 1.79)	81.0 (± 7.6)	28.92 (± 2.56)	104.0 (± 8.3)
Abrasion	20.21 (± 1.33)	101.7 (± 11.4)	32.25 (± 2.93)	104.7 (± 12.8)
MD + Abrasion	11.86 (± 2.28)	58.9 (± 3.1)	28.40 (± 1.46)	95.6 (± 6.7)

(95% confidence intervals in brackets.)

**Table 4:** Tensile properties of the woven geogrids GG40I and GG40II.

Degradation Test	GG40I		GG40II	
	T (kN·m^−1^)	E_ML_ (%)	T (kN·m^−1^)	E_ML_ (%)
Undamaged	44.36 (± 1.02)	12.1 (± 0.2)	39.83 (± 1.74)	17.6 (± 0.9)
MD	43.63 (± 3.72)	10.6 (± 0.5)	23.76 (± 2.18)	12.3 (± 1.0)
Abrasion	16.37 (± 1.25)	6.5 (± 0.5)	14.50 (± 3.60)	11.4 (± 1.0)
MD + Abrasion	14.75 (± 1.29)	6.3 (± 0.7)	7.52 (± 1.48)	10.4 (± 2.7)

(95% confidence intervals in brackets.)

**Table 5:** V*_H50_* of the nonwoven geotextiles NW300 and NW500.

Degradation Test	V*_H50_* (mm·s^−1^)	
	NW300	NW500
Undamaged	45.2 (± 6.8)	36.8 (± 3.5)
MD	47.9 (± 3.8)	36.8 (± 3.2)
Abrasion	57.3 (± 4.2)	38.6 (± 3.2)
MD + Abrasion	66.5 (± 6.8)	38.8 (± 2.4)

(95% confidence intervals in brackets.)

**Table 6:** Reduction factors obtained for the single exposures to the degradation mechanisms.

Degradation Test	W180	NW300	NW500	GG40I	GG40II
MD	2.81	1.43	1.16	1.02	1.68
Abrasion	1.16	1.16	1.04	2.71	2.75
